# Optimization of ultrasonic-assisted extraction of polysaccharides from purple glutinous rice bran (*Oryza sativa* L.) and their antioxidant activities

**DOI:** 10.1038/s41598-020-67266-1

**Published:** 2020-06-26

**Authors:** Siriluck Surin, SangGuan You, Phisit Seesuriyachan, Rattana Muangrat, Sutee Wangtueai, Anet Režek Jambrak, Suphat Phongthai, Kittisak Jantanasakulwong, Thanongsak Chaiyaso, Yuthana Phimolsiripol

**Affiliations:** 1 Division of Food and Nutrition, Faculty of Science, Chandrakasem Rajabhat University, Bangkok, 10900 Thailand; 2 Department of Marine Food Science and Technology, Gangneung-Wonju National University, Gangwon, 210-702 Republic of Korea; 3 Faculty of Agro-Industry, Chiang Mai University, Chiang Mai, 50100 Thailand; 4 College of Maritime Studies and Management, Chiang Mai University, Samuth Sakorn, 74000 Thailand; 5 Faculty of Food Technology and Biotechnology, University of Zagreb, Zagreb, Croatia; 6 Cluster of High Value Product from Thai Rice for Health, Chiang Mai University, Chiang Mai, 50100 Thailand; 7 Cluster of Agro Bio-Circular-Green Industry, Chiang Mai University, Chiang Mai, 50100 Thailand

**Keywords:** Chemical engineering, Isolation, separation and purification

## Abstract

Purple glutinous rice bran (Kum Doi Saket rice (KUM)) contains high content of edible polysaccharides and anthocyanins and has an excellent antioxidant activity. This research aimed to optimize the extraction of crude polysaccharides from defatted purple glutinous rice bran using an ultrasonic-assisted extraction (UAE) and compared with a hot water extraction (HWE). Results showed that optimal extraction condition was as follows: a defatted rice bran to water ratio of 1:20 w/v, extraction temperature and time of 70 °C for 20 min. Under the optimal extraction condition, the yield of polysaccharide of UAE (4%) was significantly higher than that obtained from the HWE (0.8%). Additionally, antioxidant activities of extracted polysaccharide including IC_50_ value DPPH, IC_50_ value ABTS, and FRAP value were 1.09 mg/mL, 2.80 mg/mL and 197 µM Fe^2+^/g, respectively. It is suggested that the UAE process is promising method to decrease the processing time and to enhance extracted polysaccharide yields by 4 times.

## Introduction

Polysaccharides have been studied for their various biological activities such as inhibiting free radicals, and antimicrobial, antitumor, anticancer, antiviral, anticoagulant and immunological activities. The biological effects of polysaccharides depend on their chemical composition, molecular weight and structure^1^. The polysaccharides extracted from edible and medicinal plants, e.g., *Pteridium aquilinum*, *Ficus carica* L., *Lycium barbarum* L., *Portulaca oleracea* L. and *Arachis hypogaea* seeds, were reported to be good sources of antioxidants^2^. Polysaccharides can inhibit free radicals generated in living organisms and protect the body tissue, thereby helping to prevent various diseases caused by tissue damage^3^. Many studies emphasized on the water-soluble polysaccharides, which are negatively charged such as with hydroxyl groups and oxygen atoms, and they can be free radical scavengers and metal chelators, leading to inhibition of lipid peroxidation^4^.

The KUM is a purple pigmented cultivar belonging to the species *Oryza sativa* L. It is widely grown in the north and northeast of Thailand and has high antioxidant activities because of its anthocyanin component^5^. The extracted polysaccharides from the defatted rice bran showed anti-cancer and/or anti-tumor properties^6^. Our previous reports showed a high potential of polysaccharides from the KUM defatted bran (KUM-DB) with antioxidant and antimicrobial activities^7^. Moreover, these polysaccharides were also modified using sulphation. The sulphated polysaccharides significantly increased immunomodulatory activity^8^.

Extraction method is important to provide biologically active compounds. The highest active compounds and low undesired materials were target in extraction. The liquid-solid extraction is commonly used to extract the required compounds. Several factors such as type of extraction, temperature, time, solvent and extracted material are key parameters. The low extraction temperature is mostly considerable because the biologically active compounds can be degraded or lost during extraction. The time-consuming of extraction is also concerned^9^. HWE is the common boiling or refluxing method using for the extraction of botanical polysaccharides. Although, HWE is a low-cost process, it uses high temperatures (95–100 °C), long extraction times and generally has a low yield^10^. The three variables significantly affected the HWE yield of *Epimedium acuminatum*. The yield was increased with an increasing ratio of water to raw material and extraction temperature from 70 to 83.5 °C, but beyond 83.5 °C, the yield was decreased with increasing extraction temperature. When the extraction time increased from 3.5 to 4 h, the yield increased significantly^11^.

The UAE has applied compared with other techniques for the extraction of bioactive compounds such as microwave-assisted extraction (MAE), supercritical fluid extraction, and enzyme-assisted extraction^12^. UAE is often inexpensive and simple. It could increase the yield of extracted components, decrease the extraction time and use lower temperatures. Therefore, it is usually used for extraction of thermolabile and unstable compounds^13^. For example, the UAE extracted polysaccharides from white button mushroom (*Agaricus bisporus*) showed that the UAE gave a higher yield than the HWE and MAE, with the UAE showing relative increases of 155 and 28%, respectively^10^. Ultrasonic has also been used to degrade polysaccharides to enhance antioxidant properties. The degraded polysaccharides did not have a different main structure but did have a reduced molecular weight^14^ and also it is very useful for extraction of polysaccharides, essential oils, proteins, peptide and pigment^15^.

Although some studies showed improvement of the extract’s properties including antioxidant activity using UAE, there is little information focusing on the crude polysaccharide extract from rice bran, especially the purple glutinous rice. Therefore, this study aimed to investigate and optimize the UAE of polysaccharide from purple glutinous rice bran cv. Kum Doi Saket (colored rice) using three independent variables including the ratio of defatted purple glutinous rice bran to water (1:1–1:3 (w/v), extraction temperature (30–70 °C) and extraction time (20–60 min) through a response surface methodology (RSM). Furthermore, it was also attempted to compare the physicochemical characteristics and antioxidant activities of polysaccharides extracted by UAE and HWE. The aim was to optimize extraction conditions while obtaining high antioxidant activities of obtained polysaccharides.

## Materials and Methods

### Materials and chemicals

Kum Doi Saket rice bran was purchased from a local grinding mill company (Organic Germinated Brown Rice Community Enterprise, Chiang Mai, Thailand) and defatted using hexane^7,8^. Chemicals used in the experiments, including 2,2-diphenyl-1-picryl-hydrazyl-hydrate (DPPH), 2,2′-azinobis-(3-ethylbenzothiazoline-6-sulfonate) (ABTS), 2,4,6-tripyridyl-s-triazine (TPTZ), 6-hydroxy-2,5,7,8-tetramethychroman-2-carboxylic acid (Trolox), sulfur trioxide–trimethylamine (STMA), anhydrous dimethyl sulfoxide (DMSO), D-glucose and ascorbic acid (vitamin C), were purchased from Sigma-Aldrich Co. Ltd. (St. Louis, MO, USA). Other reagents in this experiment were analytical grade purchased from Union Science Co., Ltd. (Chiang Mai, Thailand).

### Ultrasonic-assisted extraction (UAE) experiment

The optimal extraction condition for crude polysaccharides from KUM-DB using UAE with a static power of 150 W ultrasonic bath (S100 H, Elma Schmidbauer GmbH, Singen, Germany) was determined using RSM with Design-Expert software (version 7.0, Stat-Ease, Inc., Minneapolis, MN, USA). A Box Behnken Design was selected due to high efficient in an interation and applied to optimize the extraction variables, including the ratio of KUM-DB to water (X_1_) from 1:10-1:30 (w/v), extraction temperature (X_2_) from 30–70 °C and extraction time (X_3_) from 20–60 min. A total of 15 treatments were used (Table 1).

**Table 1:** Experimental design and properties of crude polysaccharide from KUM-DB using UAE (KUM-U).

No.	X_1_: ratio KUM-DB to water (w/v)	X_2_: extraction temperature (°C)	X_3_: extraction time (min)	Yield (%)	IC_50_ value (mg/mL)		FRAP (µM Fe^2+/^g)
					DPPH	ABTS	
1	1:10	30	40	1.61 ± 0.04 ^cd^	3.3 ± 0.6^ab^	4.9 ± 0.3^ab^	180 ± 20^b^
2	1:30	30	40	3.26 ± 0.01^b^	3.1 ± 0.2^ab^	4.1 ± 0.4^bc^	180 ± 10^b^
3	1:10	70	40	3.1 ± 0.6^b^	2.9 ± 0.1^b^	4.34 ± 0.01^b^	180 ± 10^b^
4	1:30	70	40	3.2 ± 0.7^b^	2.98 ± 0.05^b^	5.6 ± 0.4^a^	174 ± 3 ^cd^
5	1:10	50	20	2.3 ± 0.3^c^	1.8 ± 0.1^d^	3.7 ± 0.2^c^	196 ± 4^a^
6	1:30	50	20	3.5 ± 0.9^ab^	1.84 ± 0.05^d^	3.74 ± 0.01^c^	196 ± 2^a^
7	1:10	50	60	2.7 ± 0.6^bc^	5.20 ± 0.04^a^	5.11 ± 0.04^a^	170 ± 10^b^
8	1:30	50	60	3.4 ± 0.3^ab^	5.2 ± 0.1^a^	5.5 ± 0.2^a^	180 ± 3^ab^
9	1:20	30	20	1.15 ± 0.01^d^	1.2 ± 0.1^d^	2.54 ± 0.02^d^	187 ± 1^a^
10	1:20	70	20	4.3 ± 0.4^a^	1.3 ± 0.1^d^	2.8 ± 0.1^d^	200 ± 10^a^
11	1:20	30	60	3.8 ± 0.3^a^	4.2 ± 0.2^ab^	4.1 ± 0.3^bc^	180 ± 10^b^
12	1:20	70	60	3.82 ± 0.03^a^	6.0 ± 0.2^a^	4.35 ± 0.01^b^	166 ± 1^d^
13	1:20	50	40	3.9 ± 0.2^a^	2.42 ± 0.01^c^	2.5 ± 0.1^d^	180 ± 10^b^
14	1:20	50	40	3.4 ± 0.6^ab^	2.2 ± 0.2 ^cd^	2.53 ± 0.02^d^	190 ± 10^a^
15	1:20	50	40	3.4 ± 0.2^ab^	2.69 ± 0.03^c^	3.5 ± 0.1^c^	190 ± 10^a^

Data show as mean ± standard deviation (n = 3). The different letters in the same column mean significant differences (*p* ≤ 0.05).

For each treatment, 30 g of KUM-DB was put into a 3 L stainless steel bath and water was added according to the design (Table 1). After extraction, crude polysaccharide was centrifuged and evaporated to obtain concentrated supernatants. Next, the starch and protein in the supernatants were removed, and then, three volumes of 95% ethanol were added to precipitate polysaccharide. Subsequently, the precipitate was collected by centrifugation and washed with absolute ethanol. Afterwards, the precipitate was dialyzed in tap water and distilled water, respectively. Finally, the ultrasonicated crude polysaccharide (KUM-U) was dried in a vacuum oven and kept in an aluminum foil laminated polyethylene pouch at 4 °C for further analysis^7,8^.

### Comparison of the properties of crude polysaccharides from KUM-H and KUM-U

The HWE was extracted using a sample to water ratio of 1:15 (w/v) at 90 °C for 2 h done twice following the condition as described by Surin *et al*.^7,8^. In comparison, the UAE was extracted using ultrasonic extractor at KUM-DB to water ratio of 1:20 (w/v) at 70 °C for 20 min. Further processing of the extract was as stated above to obtain the hot water extract (KUM-H). The KUM-H polysaccharide was used as control and compared with optimal extraction condition of KUM-U polysaccharide.

### Determination of the physiochemical properties and anthocyanins

The physiochemical properties were evaluated as described by Surin *et al*.^7^. The yield percentage of crude polysaccharide was calculated as the ratio of crude polysaccharide per KUM-DB. The carbohydrate and protein content were evaluated using the phenol–sulfuric acid colorimetric method (D-glucose as the standard) and the Coomassie Brilliant Blue reaction method (BSA as the standard), respectively. While the starch content was measured using a total starch assay kit (AA/AMG method, Megazyme, Wicklow, Ireland).

Anthocyanin content of crude polysaccharide was measured by following the method of Settapramote *et al*.^16^ with slight modification. Briefly, an HPLC (Agilent series 1100, Waldbronn, Germany) coupled with a diode array detector was used to evaluate anthocyanin contents. The samples were dissolved in HPLC grade water (RCI-Labscan, Thailand), afterwards they were filtered through a 0.45 μm membrane disc. The prepared samples, 20 μL, were injected into a Pursuit XRs5 C18 column (250 × 4.6 mm, Agilent, USA), ambient temperature and the wavelength was 520 nm. The samples were performed with a mixture of A: 4% phosphoric acid and B: 10% acetic acid/5% acetonitrile/1% phosphoric acid in water. A gradient elution of 0–20–40% A by linear increase from 0–20–25 min.

### *In vitro* antioxidant activity

#### Sample preparation

The sample solutions were prepared by dissolving the dried powder in distilled water at final concentrations of 0.312 to 10 mg/mL. Seven methods of *in vitro* antioxidant activity were evaluated using the methods as described previously^8^. The result of DPPH radical scavenging activity, ABTS radical scavenging activity, superoxide anion scavenging activity, hydroxyl radical scavenging activity and metal chelating assay were expressed as IC_50_ values, which referred to the concentration of polysaccharide required to scavenge 50% of the radicals. Reducing power and Ferric reducing antioxidant power (FRAP) assay were also performed.

#### DPPH radical scavenging activity

The sample solution (2 mL) was mixed with 2 mL of 0.2 mmol/L DPPH solution. The mixed solution was kept in the dark at 30 °C for 30 min. The absorbance of the mixed solution was measured at 517 nm using a spectrophotometer (model UV-2101PC, Shimudzu, Kyoto, Japan) against a blank (distilled water).

#### ABTS radical scavenging activity

The sample solution was added to the ABTS radical solution with a ratio of 1:20. The mixed solution was kept at 30 °C for 6 min. The absorbance was measured at 734 nm against a blank (distilled water instead of sample solution).

#### Superoxide anion scavenging activity

The polysaccharide solution (1 mL) was added to 2.0 of mL Tris–HCl buffer (16 mM, pH 8.0) containing 76 µM NBT and 394 µM NADH. Subsequently, 0.4 mL of PMS was added, and the mixed solution was incubated at 30 °C for 5 min. The absorbance was measured at 560 nm against a blank (distilled water instead of sample solution).

#### Hydroxyl radical scavenging activity

The polysaccharide solution (1 mL) was mixed with 2 mL of 9 mmol/L FeSO_4_ solution, 2 mL of 9 mmol/L salicylic acid in 96% ethanol, and 2 mL of 8.8 mmol/L H_2_O_2_ solution. The solution was incubated at 25 °C for 60 min. The absorbance of the mixed solution was measured at 510 nm against a blank (distilled water instead of sample solution).

#### Metal chelating assay

The chelating effect of different polysaccharides on ferrous ion was measured by mixing 1 mL of samples with 0.1 mL of 2 mM FeCl_2_ and 0.2 mL of 5 mM ferrozine. The solution was kept at 30 °C for 10 min. The absorbance was measured at 562 nm against a blank (distilled water instead of sample solution). The inhibition percentage of ferrozine–Fe^2+^ complex formation was determined.

#### Reducing power

The different concentrations of samples were mixed with 2.5 mL of 0.2 M sodium phosphate buffer at pH 6.6 and 2.5 mL of 1% potassium ferricyanide [K_3_Fe(CN)_6_] solution. The mixed solution was kept at 50 °C for 20 min. Then 2.5 mL of 10% trichloroacetic acid solution was added. Then 2.5 mL of the upper layer of solution was removed and mixed with 2.5 mL of distilled water and 0.5 mL of 0.1% FeCl_3_. The absorbance of the mixed solution was measured at 700 nm.

#### FRAP assay

The sample solution (80 µL) was added to 2 mL of FRAP reagent, and the mixed solution was incubated at 37 °C for 8 min. The absorbance of the mixed solution was measured at 593 nm. A standard curve was prepared using FeSO_4_•7H_2_O solution from 100 to 1000 µM. The results were expressed as the concentrations of FeSO_4_•7H_2_O with equivalent antioxidant activity.

### Statistical analysis

Response surface methodology was applied to optimize the experimental data using Design-Expert (version 7.0, Stat-Ease, Inc., Minneapolis, MN, USA). Data were expressed as mean ± standard deviation from duplicates. A polynomial equation was fitted to the data to obtain a regression equation. And the comparison of the physicochemical properties and antioxidant activity of KUM-H and KUM-U are reported as mean ± standard deviation from triplicates. The statistical significance was evaluated using one-way analysis of variance (ANOVA) at 95% confidence.

## Results and Discussion

### Yield of crude polysaccharides

The ratio of KUM-DB to water, extraction temperature and extraction time significantly (*p* ≤ 0.05) affected the yield of the crude polysaccharides (Table 1). The regression equation of yield was a good fitting with a coefficient of determination (*R*^2^) of 0.905. The value was close to 1 indicating that the experimental and predicted values had a high degree of correlation^17^. The yields of the crude polysaccharides extracted using UAE were in the range of 1.15–4.31% (Table 1).

**Figure 1:**
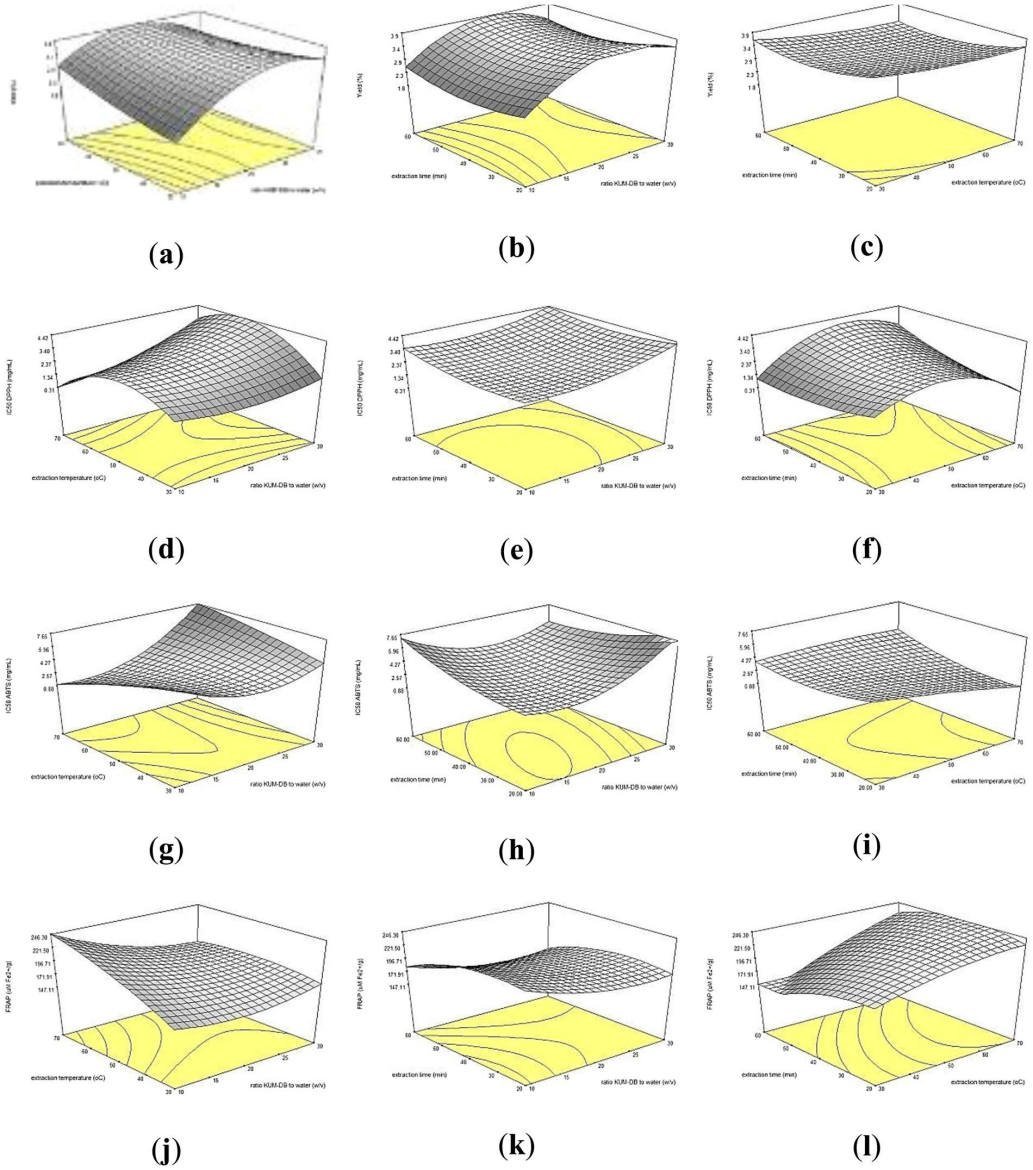
Tri-dimensional response surface contour plots showing the experimental factors: (X_1_) ratio KUM-DB to water, (X_2_) extraction temperature and (X_3_) extraction time on yield **(a–c)**, IC_50_ of DPPH assay **(d–f)**, IC_50_ of ABTS assay **(g–i)** and FRAP assay **(j–l)**.

According to the tri-dimensional response surface contour plots in Fig. 1a, the yield showed a positive correlation with ratio of KUM-DB to water and extraction temperature. At all extraction temperatures, increasing the ratio of KUM-DB to water had a positive impact in the range of 1:10 to 1:25 w/v but it gave slightly lower yield, when the ratio of KUM-DB to water was greater than 1:25 w/v. The result is in agreement with the study previously of Cheung and Wu^18^. They reported that the ratio of material to water and extraction temperature (70–80 °C) affected the polysaccharide yield from *Zizyphus jujuba* cv. *jinsixiaozao* extracted by UAE improving the mass transfer rates. In addition, the yield of crude polysaccharides was rapidly increased when increasing the extraction temperature and the highest yield was observed at 60–70 °C. Using lower extraction temperatures (below 40 °C) could not disrupt the cell wall causing to obtain a low yield of polysaccharides from rice bran.

The tri-dimensional response surface contour plots of the ratio of KUM-DB to water (X_1_) with the extraction time (X_3_) on the yield of crude polysaccharides are shown in Fig. 1b. The yield of crude polysaccharides rapidly increased with increasing the ratio of KUM-DB to water from 1:15 to 1:20 w/v but the yields were slightly decreased when the ratio of KUM-DB to water was higher than 1:20 w/v. For the extraction time, the yield increased with increasing extraction time. Overall, the UAE increase the yield of polysaccharides because it can disrupt the cell wall of plant tissues, resulting in the polysaccharides easily released into the water at the early period of extraction^19^.

Figure 1c shows the effect of extraction temperature and extraction time on the crude polysaccharides yield at a fixed the ratio of KUM-DB to water of 1:20 w/v. For the extraction temperature, the crude polysaccharides yield was slightly increased with increasing temperature and had the highest yield at 70 °C. For extraction time, the crude polysaccharides yield decreased with increased extraction time. The longer extraction time led to degrade the polysaccharides into free sugars, resulting in a decrease of polysaccharides yield^12,20^.

### *In vitro* antioxidant activity

#### Effect of UAE on DPPH radical scavenging activity of crude polysaccharides

The DPPH assay has been widely used as a tool for evaluating the free radical scavenging ability of antioxidant agents^10^. Crude polysaccharides could inhibit free radicals to become a stable molecule by donating electrons or hydrogen atom to the free radical^21^. The regression equation of DPPH radical scavenging activity was obtained by fitting the quadratic polynomial equation with the data. The effect of extraction on the IC_50_ DPPH scavenging activity is shown in Table 1. It was found that only extraction time (X_3_) had significantly (*p* ≤ 0.05) affected the IC_50_ value of DPPH scavenging activity. The interaction effects of extraction time (X_3_) with ratio of KUM-DB to water (X_1_) and with extraction temperature (X_2_) on the IC_50_ DPPH scavenging activity are also shown in Fig. 1e,f, respectively. As shown in Fig. 1f, the extraction time had a positive effect on the IC_50_ DPPH scavenging activity. These results showed that the lower IC_50_ DPPH value was observed when extraction time was shorter than 30 min. The lower IC_50_ DPPH value corresponds to a higher potential to inhibit free radicals^22^. When extracting for long time, the IC_50_ DPPH value increased. However, the ratio of KUM-DB to water and extraction temperature had no significant (*p* > 0.05) effect on the IC_50_ DPPH value (Fig. 1d).

#### Effect of UAE on ABTS radical cation scavenging activity of crude polysaccharides

The ABTS assay is often used to estimating total antioxidant power^7^. From the regression equation, it was found that the extraction time (X_3_) was the most significant factor affecting the IC_50_ value of ABTS as shown in Table 2. The extraction time had a negative effect on the antioxidant activity. The IC_50_ value of ABTS increased when extracted for longer times. The higher value of IC_50_ ABTS corresponds to lower activity. Therefore, the greatest IC_50_ value of ABTS scavenging activity of crude polysaccharides was found when the extraction time was shorter than 30 min (Fig. 1h,i). The mechanism to explain this, is that the plant cell wall was disrupted by the collapse of cavitation bubbles, which were near a cell walls which increased the release of the crude polysaccharide. With longer extraction times, the polysaccharides were noticeably degraded to shorter chains and some functional groups were cut^23^. Therefore, using the shorter extraction time is potentially beneficial for the inhibition of free radicals. The ratio of KUM-DB to water and extraction temperature had no significant (*p* > 0.05) effect on the IC_50_ value for ABTS radical scavenging activity (Fig. 1g).

**Table 2:** Regression equations, coefficient of determinations (*R*^2^), and *p*-values of each response of crude polysaccharides from KUM-DB using UAE (KUM-U).

Responses	Regression equation	Adj. *R*^2^	*p-value*
Yield (%)	−8.31 + 0.33 X_1_ + 0.16 X_2_ + 0.14 X_3_ − 1.84 × 10^−3^X_1_X_2_ − 1.02 × 10^−3^ X_1_X_3_ − 1.80 × 10^−3^ X_2_X_3_ − 4.04 × 10^−3^X_1_^2^ − 2.43 × 10^−4^X_2_^2^ − 1.64 × 10^−4^X_3_^2^	0.905	0.0036
IC_50_ DPPH value (mg/mL)	5.97 − 0.21 X_1_ − 0.08 X_2_ + 0.08 X_3_ + 2.75 × 10^−4^X_1_X_2_ − 1.00 × 10^−4^ X_1_X_3_ + 1.06 × 10^−3^ X_2_X_3_ − 4.99 × 10^−3^ X_1_^2^ + 3.79 × 10^−4^X_2_^2^ + 1.44 × 10^−3^X_3_^2^	0.909	0.0033
IC_50_ ABTS value (mg/mL)	12.9 − 0.73 X_1_ − 0.15 X_2_ + 6.07 × 10^−3^X_3_ + 2.61 × 10^−3^ X_1_X_2_ + 3.90 × 10^−4^X_1_X_3_ + 4.71 × 10^−5^X_2_X_3_ + 0.01 X_1_^2^ + 1.03 × 10^−3^ X_2_^2^ + 4.32 × 10^−4^X_3_^2^	0.889	0.0052
FRAP value (µM Fe^2+^/g)	140.3 + 1.11 X_1_ + 2.16 X_2_ − 0.53 X_3_ − 1.00 × 10^−2^X_1_X_2_ + 0.01 × _1_X_3_ −0.01 X_2_X_3_ − 0.02 X_1_^2^ − 0.01 X_2_^2^ + 6.25 × 10^−3^X_3_^2^	0.914	0.0029

*p*-value indicates a significant difference at 95% confidence (*p* ≤ 0.05). X_1_ = ratio of KUM-DB to water (w/v), X_2_ = extraction temperature (°C), X_3_ = extraction time (min).

#### Effect of UAE on ferric-reducing activity power of crude polysaccharides

The ferric-reducing activity power (FRAP) assay is another assay to evaluate the antioxidant activity of crude polysaccharides. This method measures the ability of a sample to reduce the TPTZ-Fe(III) complex to the TPTZ-Fe(II) complex^24^. The crude polysaccharides were in the range of 166–200 µM Fe^2+^/g (Table 1). The regression equations with FRAP were obtained by fitting the quadratic polynomial equation with the data, as shown in Table 2. The results showed that extraction time was a negative factor on the FRAP of crude polysaccharides. The higher values of FRAP correspond to higher activity. The tri-dimensional plot of the interaction between extraction time (X_3_) with the ratio of KUM-DB to water (X_1_) and with extraction temperature (X_2_) showed the quadratic effect on FRAP of crude polysaccharides are shown in Fig. 1j–1, respectively. Figure 1k shows that the FRAP value increased with shorter extraction times. The similar results of the interaction between extraction time (X_3_) and extraction temperature (X_2_) are presented in Fig. 1.

### Optimization and validation of ultrasonic-assisted extraction condition

**Figure 2:**
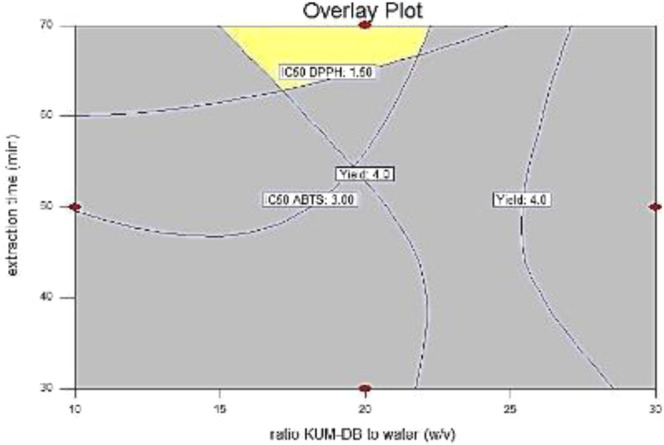
Overlay plot of the response surface regression model between the ratio of KUM-DB to water and extraction temperature; fixed extraction time at 20 min.

The optimization of UAE application aimed to provide the highest yield and the greatest antioxidant activities, the lowest IC_50_ DPPH and IC_50_ ABTS values and the highest FRAP value. Optimizing condition for UAE using response surface is shown in Fig. 2. The optimal condition for UAE was a ratio of KUM-DB to water of 1:20 w/v, extraction temperature of 70 °C and extraction time of 20 min. With optimal condition the highest yield of crude polysaccharides (4.04%) was close to the predicted value of 4.34%. Antioxidant activities including IC_50_ DPPH, IC_50_ ABTS, and FRAP value were 1.09 and 2.80 mg/mL and 197 µM Fe^2+^/g, respectively. The predicted and experimental values were calculated for the percentage of approximated error which should not be above 10% of the proximity error. The percentage of approximated error was in the range of 0.38–7.55. This indicated that the results of the validation were in acceptable agreement between the predicted and experimental values.

### Properties of crude polysaccharides of KUM-U and KUM-H

#### Chemical properties of crude polysaccharides

The KUM-U (4.1%) gave significantly (*p* ≤ 0.05) higher yield than KUM-H (0.86%). Previous reports found that the extraction of crude polysaccharides using UAE for increasing productivity could accelerate the extraction time, use less power, and lower the extraction temperature^19^. The comparison of HWE, microwave-assisted extraction (MAE) and UAE to extract crude polysaccharides from white button mushroom (*Agaricus bisporus*) showed that extraction of crude polysaccharides using UAE had the highest yield by about 155 and 28% when compared to HWE and MAE, respectively^10^.

The chemical compositions including total carbohydrate, protein, total starch and anthocyanin content of the KUM-H and KUM-U are presented in Table 3. The total carbohydrate and total starch content of the KUM-U were significantly (*p* ≤ 0.05) higher than those of the KUM-H. However, protein and anthocyanin content of the KUM-U were significantly (*p* ≤ 0.05) lower than those of the KUM-H. Previously research of Li *et al*.^25^ also found that using UAE for extracting polysaccharides from *Zizyphus jujube* cv. *jinsixiaozao* showed lower content of protein than conventional extraction. Because the higher temperature and longer time of HWE increased the dissolving of proteins in water, the lower temperature and shorter time of UAE increased the purity of the crude polysaccharides. Earlier report of Shang *et al*.^26^ revealed that the polysaccharide yield of UAE (9.43%) was higher than that of HWE (8.35%), the extraction time of UAE was significantly reduced when compared to HWE. Again, the yield of polysaccharides increased in the first 10 min and decreased when increased of extraction time^27^. This is probably due to degradation of polysaccharides by longer time^28^ and higher temperature of UAE^29^. Generally, anthocyanins were entrapped in the matrix or formed hydrogen linkages between hydroxyl groups of polyphenols and the oxygen atom of the glycosidic linkages of polysaccharides, but the anthocyanins could be dissociated from polysaccharides by the cavitation force using UAE and became free molecules in water^19^. Therefore, the anthocyanin content of the KUM-U was lower than that of the KUM-H.

**Table 3:** Chemical properties of crude polysaccharide extracted using hot water (KUM-H) and ultrasonic–assisted extraction (KUM-U).

Properties	KUM-H	KUM-U
Yield (%)	0.86 ± 0.12^b^	4.0 ± 0.1^a^
Total carbohydrate (%)	79 ± 3^b^	97 ± 1^a^
Protein (%)	19.6 ± 0.3^a^	3.9 ± 0.1^b^
Total starch (%)	1.2 ± 0.1^b^	7.4 ± 0.3^a^
Anthocyanin content (mg/g)	0.28 ± 0.02^a^	0.12 ± 0.01^b^

The different letters in the same row mean significant differences (*p* ≤ 0.05).

#### Scavenging activity of DPPH and ABTS radicals

The ability of both crude polysaccharides to inhibit DPPH radicals increased when concentration increased, indicating that the KUM-H and KUM-U could donate electrons or hydrogen to DPPH radicals. The IC_50_ value of the KUM-H and KUM-U were not significantly different (*p* > 0.05) as presented in Table 4. This result showed similarly with IC_50_ value of polysaccharide wild garlic (*Allium ursinum* L.), which it had 0.73^23^. For ABTS testing, the crude polysaccharides of KUM-H and KUM-U had strong scavenging ability of the ABTS radical in a concentration-dependent manner, indicating that the KUM-H and KUM-U might act as an electron or hydrogen donator to scavenge ABTS radicals. The IC_50_ values of KUM-H and KUM-U were not significantly different (*p* > 0.05), at 2.93 and 2.82 mg/mL, respectively (Table 4).

**Table 4:** Antioxidant activities of crude polysaccharide extracted using hot water (KUM-H) and ultrasonic –assisted extraction (KUM-U).

Antioxidant activities	KUM-H	KUM-U
IC_50_ DPPH (mg/mL) ^ns^	0.94 ± 0.03	1.05 ± 0.01
IC_50_ ABTS (mg/mL) ^ns^	2.93 ± 0.01	2.82 ± 0.03
IC_50_ Superoxide radical (mg/mL)	1.5 ± 0.1^b^	4.8 ± 0.4^a^
IC_50_ Hydroxyl radical (mg/mL)	1.04 ± 0.01^b^	1.47 ± 0.01^a^
IC_50_ Metal chelating (mg/mL)	1.25 ± 0.04^b^	3.7 ± 0.4^a^
Reducing power (mg/mL)	4.6 ± 0.1^b^	6.4 ± 0.3^a^
FRAP value (µmol Fe^2+^/g)^ns^	199 ± 5	200 ± 5

Data show mean ± standard deviation (n = 3). The different letters in the same row mean significant difference (*p* ≤ 0.05). ^ns^means non-significant difference (*p* > 0.05).

#### Scavenging activity for superoxide anion radicals

The scavenging activity IC_50_ of crude polysaccharides for superoxide radicals were of KUM-H and KUM-U were significantly different (*p* ≤ 0.05), at 1.54 and 4.85 mg/mL, respectively (Table 4). Thus, the KUM-H had higher scavenging activity for the superoxide anion radical. The IC_50_ of superoxide anion radicals from extracted polysaccharide of the *Curcuma phaeocaulis* Rhizomes by HWE (0.36 mg/mL) was also greater than UAE (0.51 mg/mL)^13^. However, both crude polysaccharides were effective scavengers of the superoxide anion radical. They might be able to prevent the dismutation reaction, which produces the other reactive oxygen species such as hydrogen peroxide, hydroxyl radical, and singlet oxygen from the superoxide anion radical. The superoxide anion radical and its derivatives could induce oxidative damage in lipids, proteins and DNA which is extremely harmful to human living^30^.

#### Scavenging activity for hydroxyl radicals

It was found that the KUM-H had higher scavenging ability for hydroxyl radicals than KUM-U (*p* ≤ 0.05) because the KUM-H had lower IC_50_ values than the KUM-U, 1.04 and 1.47 mg/mL, respectively (Table 4). This result conformed to the polysaccharide extraction of *Flammulina velutipes*. They reported the hydroxyl radicals scavenging activities of polysaccharide concentration at 2.50 mg/mL, HWE (36.87%) was higher efficiency than UAE (31.21%)^31^. However, both polysaccharides could inhibit hydroxyl radicals, which occur in the body, to protect against cell death. Hydroxyl radicals are considered to be a highly potent oxidant, which can react with most biomolecules in living cells and induce severe tissue damage or cell death^32^.

#### Scavenging activity for metal chelating

The ability to chelate metals of both crude polysaccharides were concentration-dependent from 0.312 to 10 mg/mL. The IC_50_ values of KUM-H and KUM-U were 0.94 and 1.05 mg/mL, respectively. This result was also in agreement with Zhang *et al*.^31^ who found that the chelating capability of HWE was more effective than UAE. The KUM-H had a significantly better (*p* ≤ 0.05) capability to chelate metals (Table 4). Metal ions accelerate lipid oxidation by breaking down hydrogen and lipid peroxidases to reactive free radicals using the Fenton reaction. Therefore, transition metal chelating activity is important in reducing lipid peroxidation^32^.

#### Reducing power

The reducing power values (Table 4) indicated that the reducing power values of the KUM crude polysaccharides were significantly different (*p* ≤ 0.05). The concentration of KUM-H and KUM-U needed for an absorbance of 0.5 at 700 nm were 4.57 and 6.38 mg/mL, respectively. The results suggested that hot water extraction was suitable to extract polysaccharides from the KUM rice bran, resulting in the highest reducing power and higher potential hydrogen-donating ability. The reducing power assay is often used as an indicator of electron-donating activity to reduce Fe^3+^ to Fe^2+^ by donating an electron. The reducing ability of polysaccharides is an indicator of potential antioxidant properties and the increasing absorbance indicates an increase in reducing power^33^. Zhao *et al*.^34^ also found that the reducing power of polysaccharide from *L*. *japonica* by HWE was only 0.12 at 1.84 mg/mL, while our polysaccharide extracts reached 0.5 at 4.57 mg/mL.

#### Ferric reducing antioxidant power

The FRAP measures compounds as reductants in a redox linked colorimetric reaction and the value reflects the reducing power of antioxidants. The antioxidant potentials of different samples are estimated by their ability to reduce the TPTZ–Fe(III) complex to the TPTZ–Fe(II) complex with an maximum absorption at 593 nm. The reduction of absorbance is proportional to the antioxidant content^35^. The FRAP value of the KUM-H and KUM-U were not significantly different (Table 4) at about 200 µmol Fe^2+^/g. The antioxidant capacities of both crude polysaccharides were excellent. Another study from Jia *et al*.^36^, they reported that their polysaccharides from Hawk tea (*Litsea coreana* var. *lanuginosa*) had FRAP value in range 0.9–2.1 mmol/L at the concentration of 1.25 mg/mL.

Overall, the crude polysaccharides extracted from the KUM-DB had a potential to scavenge free radicals and had reducing power. The antioxidant abilities of crude polysaccharides are influenced by monosaccharide composition, molecular weight, degree of branching, and conformation^37^. Moreover, the phenolic compound in polysaccharides in the form of polysaccharides-phenolic complexes or phenolic compounds also affect antioxidant properties^38^. The antioxidant activity of polysaccharides from KUM-DB, which is a purple rice, was higher than the polysaccharides from white rice bran. Considering several antioxidant activities, the concentration of polysaccharides from KUM-H in IC_50_ DPPH, IC_50_ metal chelating and reducing power were lower than the concentration of polysaccharides needed from white rice^39^ (excepted the reducing power). Therefore, the present study showed 98% and 37.5% higher activity for IC_50_ DPPH and IC_50_ metal chelating, respectively, for purple versus white rice. Again, using hot water extraction to extract polysaccharides from medicinal mushrooms containing phenolic compound showed a high linear correlation between the scavenging activity of the polysaccharides and their content of phenolic compounds. Extraction of crude polysaccharides with HWE and ethanol precipitation is not sufficient to remove the phenolic compound from the crude polysaccharides^40^. Therefore, the KUM-H had a better antioxidant capability than the KUM-U as shown in the chromatogram (Figs. 1S and 2S). This is probably due to the higher anthocyanin content of the KUM-H as synergistic effect with polysaccharide^38^.

## Conclusions

The optimum condition of UAE was determined as follows: a ratio of defatted rice bran to water of 1:20 w/v, extraction temperature of 70 °C, and extraction time of 20 min. This optimal extraction condition gave the highest yield of crude polysaccharides (4.04%), which was closed to the predicted value. Moreover, antioxidant activities including IC_50_ value DPPH, IC_50_ value ABTS, and FRAP value were 1.09 mg/mL, 2.80 mg/mL and 197 µM Fe^2+^/g, respectively. The physical properties and antioxidant activities of KUM-H and KUM-U were compared in this work. Results showed that percentage yield of KUM-U was approximately 4 times higher than that of KUM-H. It is suggested that UAE could be a suitable process to extract crude polysaccharides with high yield, shorter processing time and acceptable antioxidant activities. Future works are required to investigate and characterize obtained polysaccharides in terms of structure and interaction effects of polysaccharide and phenolic compounds using advanced measurement.

## Supplemenatry information

Supplemenatry information.
