# Supplementary material for: Optimization of ultrasonic-assisted extraction of polysaccharides from purple glutinous rice bran (Oryza sativa L.) and their antioxidant activities
Source: Sci Rep. 2020 Jun 26;10:10410. doi: 10.1038/s41598-020-67266-1 (PMC7319984; doi:10.1038/s41598-020-67266-1)
Supplement: Supplementary file 1 — Supplemenatry information. [file 41598_2020_67266_MOESM1_ESM.docx]

**Optimization of ultrasonic-assisted extraction of polysaccharides from**

**purple glutinous rice bran (*Oryza sativa* L.) and their antioxidant activities**

Siriluck Surin^1^, SangGuan You^2^, Phisit Seesuriyachan^3,7^, Rattana Muangrat^3^, Sutee Wangtueai^4^,

Anet Režek Jambrak ^5^, Suphat Phongthai^3,6,^ Kittisak Jantanasakulwong^3,7^,

Thanongsak Chaiyaso^3,7^ and Yuthana Phimolsiripol^3,6,7*^

*^1^ Division of Food and Nutrition, Faculty of Science, Chandrakasem Rajabhat University, Bangkok, 10900 Thailand*

*^2^ Department of Marine Food Science and Technology, Gangneung-Wonju National University, Gangwon, 210-702 Republic of Korea*

*^3^ Faculty of Agro-Industry, Chiang Mai University, Chiang Mai, 50100 Thailand*

*^4^ College of Maritime Studies and Management, Chiang Mai University,*

*Samuth Sakorn, 74000 Thailand*

*^5^ Faculty of Food Technology and Biotechnology, University of Zagreb, Zagreb, Croatia*

*^6^ Cluster of High Value Product from Thai Rice for Health, Chiang Mai University,*

*Chiang Mai, 50100 Thailand*

*^7^ Cluster of Agro Bio-Circular-Green Industry, Chiang Mai University,*

*Chiang Mai, 50100 Thailand*

**Correspondence Author:** Y. Phimolsiripol

E-mail: [yuthana.p@cmu.ac.th](mailto:yuthana.p@cmu.ac.th) Tel. +66 53948236 Fax. +66 53948230

**Running title:** Purple glutinous rice bran polysaccharides

**Supplementary Figures**


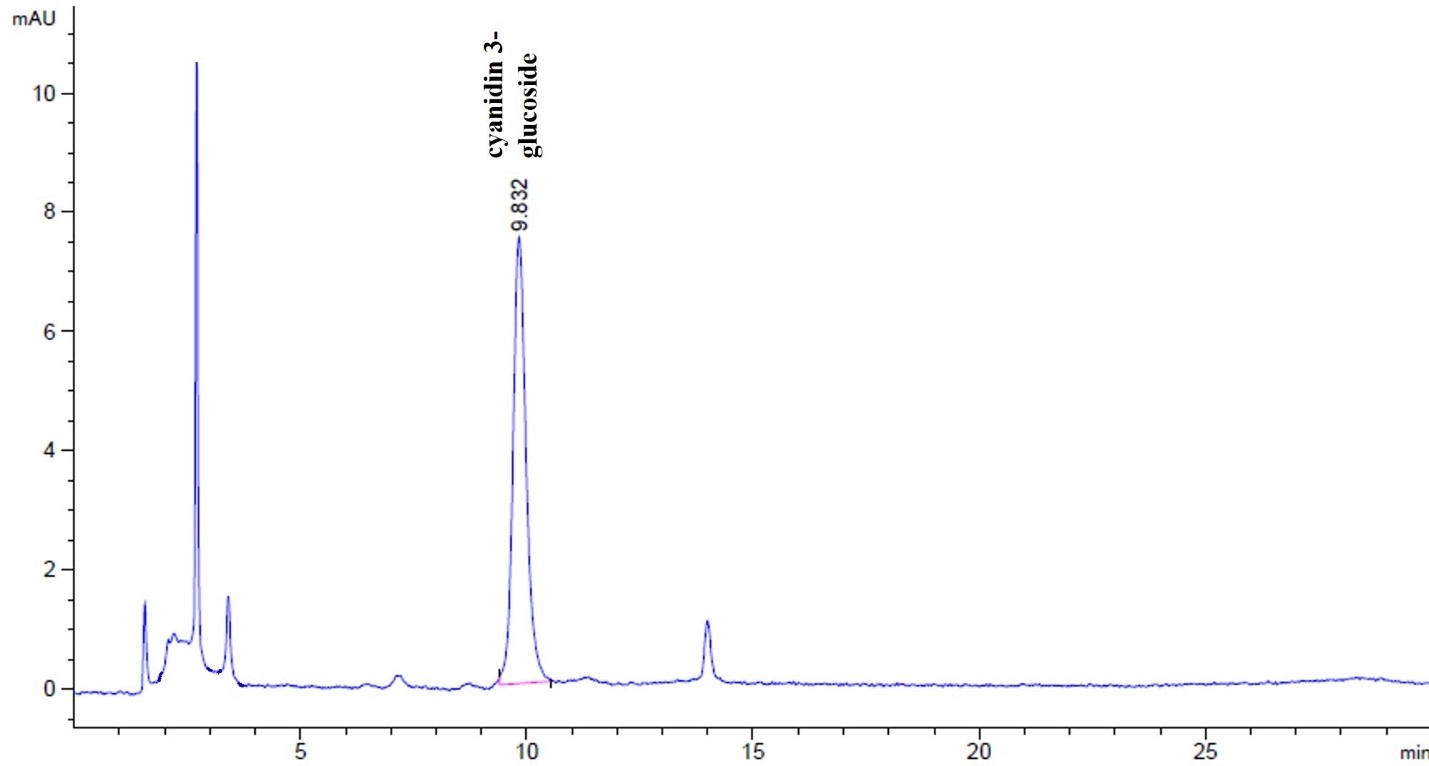


Fig. 1S. HPLC chromatogram of anthocyanin contained in polysaccharide extracted by HWE


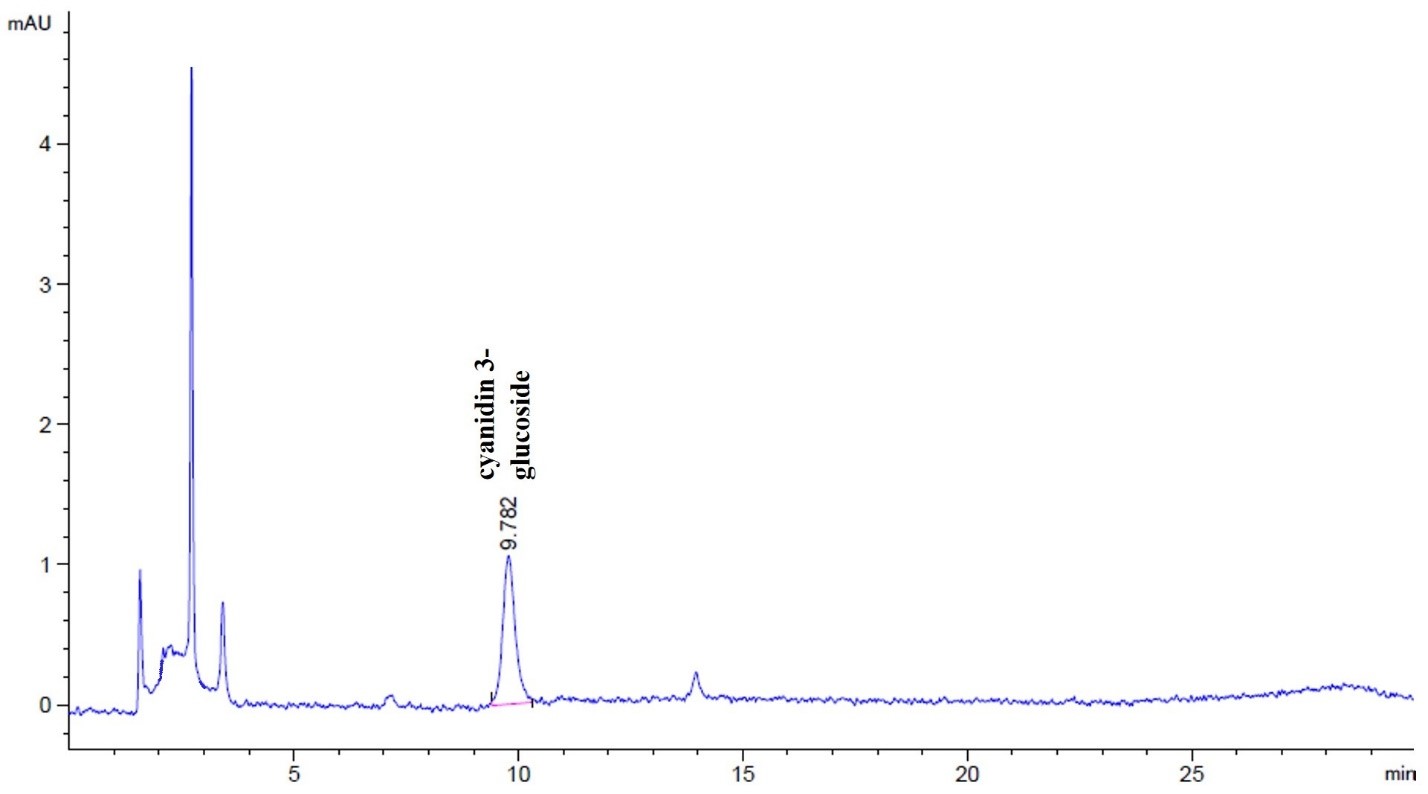


Fig. 2S. HPLC chromatogram of anthocyanin contained in polysaccharide extracted by UAE.
